# The dark side of PLK1: Implications for cancer and genomic instability

**DOI:** 10.18632/oncotarget.28456

**Published:** 2023-06-27

**Authors:** Lilia Gheghiani, Zheng Fu

**Keywords:** polo-like kinase 1 (PLK1), oncogene, chromosomal instability, cell cycle checkpoints, tumorigenesis

**Figure 1:**
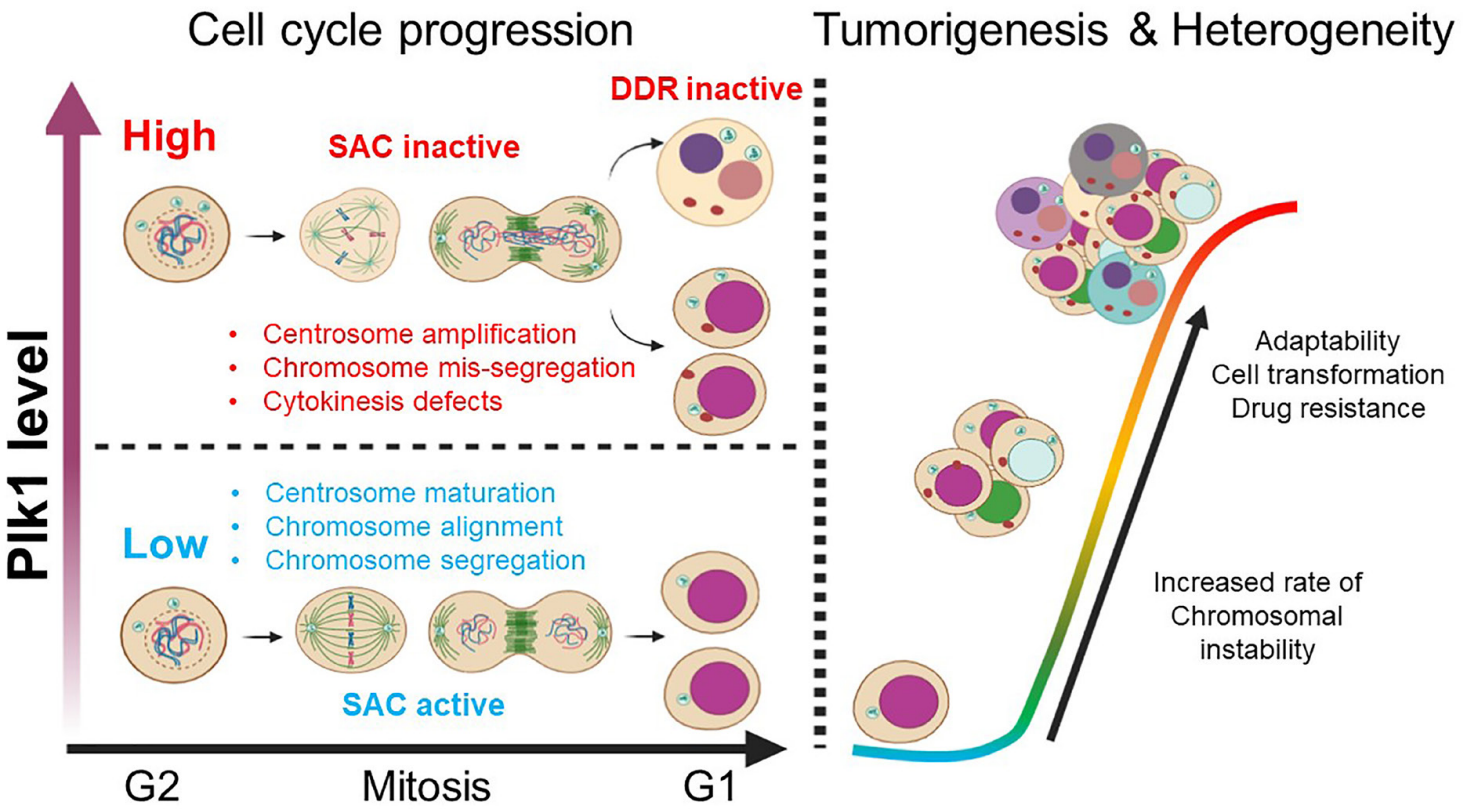
The role of PLK1 in tumorigenesis and cancer heterogeneity. Left: High level of PLK1 induces a multitude of mitotic defects and inactivation of cell cycle checkpoints (Abbreviations: SAC: spindle assembly checkpoint and DDR: DNA damage response), leading to uncontrolled cell proliferation and accumulation of chromosomal instability (CIN). Right: PLK1 overexpression leads to increased CIN, which in turns promotes checkpoint adaptation, cell transformation, drug resistance, and tumor heterogeneity.

PLK1 is a master regulator of the cell cycle that has functions ranging from mitotic commitment, centrosome maturation, bipolar spindle formation, chromosome segregation, to furrow formation in cytokinesis, together preventing genomic instability and the transmission of altered DNA to daughter cells [1, 2] (Figure 1). Beside its role during mitosis, PLK1 is also a modulator of DNA replication, DNA damage response (DDR), G2 DNA-damage checkpoint, chromosome dynamics, and microtubule dynamics by its interaction with and phosphorylation of several key factors involved in these pathways [3, 4]. The coordination of PLK1 functions at various stages of the cell cycle relies on a spatial and temporal regulation mainly via transcriptional and post-translational modifications [2, 5, 6]. PLK1 expression patterns are under dynamic control and related to cell cycle progression in normal adult tissues [6, 7]. Usually low in interphase, PLK1 protein levels increase gradually throughout the S phase and reach a maximum in the G2/M phase. They are then largely degraded after mitosis [4, 5, 7]. PLK1 expression (at both mRNA and protein level) is found upregulated in actively dividing tumor cells compared to normal cells [3, 8, 9]. This increased expression is a common feature of human cancer, manifesting in a variety of human tumors, including melanoma, carcinomas (head and neck squamous cell carcinoma, esophageal squamous cell carcinoma, hepatocellular carcinoma, gastric carcinoma, and prostate carcinoma), sarcomas, and lymphomas [9–13]. Moreover, upregulation of PLK1 is associated with high tumor grade and poor patient prognosis [9]. More importantly, downregulation of PLK1 expression usually results in decreased proliferation, mitotic arrest, and apoptotic cell death of various cancer cells with no or minimal effect on normal cells [7, 14–16], suggesting that PLK1 may be a potential biomarker to predict cancer aggressiveness and an attractive target for cancer therapeutics. Consistently, an extensive body of literature suggest that PLK1 is directly involved in tumor initiation and progression [3, 8, 17]. Several PLK1 small molecule inhibitors have reached clinical trials. Even though studies have suggested that PLK1 contributes to tumorigenesis, the ability of PLK1 to drive oncogenic transformation on its own in *vivo* was still questionable due to a lack of sophisticated animal models for experimentation [18, 19].

To address this important scientific question, we generated a new genetically engineered mouse (GEM) model using the CAGGS (cytomegalovirus (CMV) early enhancer/chicken β-actin) promoter to drive exogenous PLK1 expression, allowing its ubiquitous and robust gene expression in transgenic mice [20]. Mice were intercrossed to generate cohorts of wild-type (WT), *Plk1*^TA/+^ (heterozygous for the activated *Plk1* transgene), and *Plk1*^TA/TA^ (homozygous for the transgene) in order to have a graded expression of the *Plk1* transgene in a wide variety of tissues and organs. Our transgenic model mirrors the magnitude of enhanced PLK1 expression observed in human tumors. Ubiquitously enhanced expression of PLK1 was sufficient to drive spontaneous tumorigenesis in multiple tissues, providing the first evidence that PLK1, when overexpressed, becomes a potent oncogene *in vivo*. Using fluorescence *in situ* hybridization, karyotyping, and real-time live-cell imaging experiments, we have demonstrated that increased PLK1 expression caused multiple defects in mitosis and cytokinesis, which drives chromosomal instability (CIN). We monitored cell cycle progression of primary pulmonary alveolar epithelial cells and mouse embryonic fibroblasts derived from these transgenic mice and showed that PLK1 overexpression promoted the formation of supernumerary centrosomes, leading to multipolar mitotic spindle assembly, merotelic kinetochore-microtubule attachment, lagging chromosomes, chromatin bridges, and cytokinesis failures (Figure 1). The genomic chaos elicited by increased PLK1 expression failed to halt cell cycle progression, resulting in binucleated cells, giant multinucleated cells, and micronucleated cells (Figure 1). How do these cells tolerate genomic chaos? Further study showed that PLK1 overexpression override cell cycle checkpoints (both spindle assembly checkpoint (SAC) and DNA damage checkpoint). PLK1 upregulation impaired key regulators of the SAC that monitors kinetochore-microtubule attachments and ensures correct segregation of chromosomes. Furthermore, DNA damage lesions were detected, as evidenced by the activation (phosphorylation) of the ataxia-telangiectasia mutated (ATM) kinase in tumor tissue derived from *Plk1* transgenic mice; however, the propagation of the DNA-damage signals through the ATM-checkpoint kinase 2 (Chk2) pathway and p53-mediated checkpoint were impaired when the level of PLK1 is high. These provide an explanation of how chromosomally unstable *Plk1* transgenic cells continue to proliferate and accumulate CIN, leading to malignant transformation and cancer development. A deeper understanding of the mechanism of action by which PLK1 compromises these checkpoints would guide the development of more effective and targeted treatment regimens for cancer patients with PLK1 overexpression.

In summary, this study provides a novel GEM model that recapitulates the increased PLK1 expression observed in many human cancers and demonstrates that PLK1 overexpression drives spontaneous tumor formation in multiples organs in mouse, revealing the dark side of PLK1 as a potent proto-oncogene. CIN is a hallmark of human cancer, but, in some cases, CIN has also been associated with tumor cell death depending of the degrees and sites of CIN [21–23]. Multiple lines of evidence provided by this study strongly support PLK1 as a CIN gene, which may open a new avenue to target CIN-positive cancers in humans. Currently, the clinical utility of PLK1 inhibitors has yet to be realized due to dose-limiting toxicities, poor efficacy as a monotherapy, and a lack of predictive biomarkers to measure the response to PLK1 inhibition [14, 16, 24]. Alternative therapeutic strategies, such as co-delivery systems using nanoparticles or combination therapies, are under development in order to enhance the efficacy of PLK1 inhibition [25–28]. With expanding discoveries of PLK1 function and mechanisms of action, we hope that PLK1-targeted therapies will soon join the frontlines in the fight against cancer.
